# RNAi-based functional analysis of a theta-class glutathione S-transferase implicated in deltamethrin detoxification in *Pardosa astrigera* (Araneae: Lycosidae)

**DOI:** 10.3389/fphys.2025.1693654

**Published:** 2025-10-15

**Authors:** Rui Li, Liyalin Jiao, Shuxin Zhai, Xingjie Jin, Congcong Cui, Boqi Ren, Xinghua Zhang, Fangyu Shen, Min Ma, Michael A. Rieger, Xinmin Li

**Affiliations:** ^1^ College of Plant Protection, Shanxi Agricultural University, Jinzhong, China; ^2^ Shanxi Key Laboratory of Integrated Pest Management in Agriculture, Shanxi Agricultural University, Jinzhong, China; ^3^ Technology Center for Genomics and Bioinformatics, Department of Pathology and Laboratory Medicine, University of California, Los Angeles, CA, United States

**Keywords:** Pardosa astrigera, glutathione S-transferase, detoxification, deltamethrin, RNAi

## Abstract

**Introduction:**

Glutathione S-transferases (GSTs) play a critical role in insecticide detoxification. This study investigates the molecular characteristics and biological function of a theta-class glutathione S-transferase gene, PaGSTt1, in the spider Pardosa astrigera.

**Methods:**

Based on transcriptome data, the PaGSTt1 gene (GenBank accession number: PV848051.1) was cloned using RT-PCR and characterized via bioinformatics analysis. Its expression patterns across different developmental stages (2nd to 6th instar nymphs and adults), tissues (cephalothorax, abdomen, and legs), and in response to deltamethrin exposure (LC_10_, LC_30_, LC_50_) were quantified by RT-qPCR. RNA interference (RNAi) was employed to knock down PaGSTt1 in male spiders, and the subsequent change in susceptibility to deltamethrin was evaluated.

**Results:**

The PaGSTt1 gene contains a 678 bp open reading frame encoding 225 amino acids. Expression was detected at all developmental stages, with the highest level in 5th instar nymphs. In adults, expression was highest in the abdomen and was generally higher in males than in females. Deltamethrin exposure significantly induced PaGSTt1 expression: at LC_10_ (5.151 mg/L), upregulation occurred at 6 h and 48 h; at LC_30_ (8.619 mg/L), induction was observed at 48 h; and at LC_50_ (12.311 mg/L), significant upregulation was detected at 6 h, 24 h, and 48 h. RNAi successfully silenced PaGSTt1, reducing its expression by 78.69% after 24 hours. This knockdown led to a 34.54% increase in mortality in male spiders exposed to LC_30_ deltamethrin compared to the dsGFP control group.

**Discussion:**

The findings demonstrate that PaGSTt1 is inducible by deltamethrin and that its knockdown significantly increases mortality in P. astrigera, indicating its important role in the detoxification of this insecticide. This suggests that PaGSTt1 contributes to pesticide tolerance mechanisms in this species.

## 1 Introduction

In agricultural production, chemical control has consistently been the primary measure for pest management due to its rapid effectiveness and high efficiency. However, the overuse of insecticides has also led to certain negative impacts, such as environmental pollution, disruption of natural pest control by natural enemies, and ecological imbalance. Arachnids, the second-largest group of arthropods after insects, are globally distributed and play critical roles in ecological food webs and agroecosystems. Among them, *Pardosa astrigera* (Araneae: Lycosidae), a species of wolf spider, is an active, ground-dwelling predator widely found in East Asian agroecosystems. It is particularly abundant in wheat, cotton, corn, and rice fields across the Yangtze and Yellow River Basins in China (Gao et al., 2022). *Pardosa astrigera* possesses several advantageous ecological traits that make it a dominant natural enemy species: it has a high reproductive rate, wide prey spectrum, strong tolerance to starvation, and rapid movement especially in males (Li and Zhang, 2022; Zhai et al., 2025). However, the widespread use of synthetic insecticides has posed a significant threat to *P. astrigera*. Insecticides not only promote pest resistance but also directly or indirectly harm non-target organisms, including beneficial arthropods such as spiders (Li, 2017; Enayati et al., 2005). Long-term exposure to insecticides like deltamethrin can impair the physiology, behavior, and survival of spiders, reducing their pest control efficacy and endangering their long-term persistence in fields (Li and Zhang, 2022; Sheehan et al., 2001). Therefore, investigating molecular detoxification mechanisms of spiders under insecticide stress is of great significance for assessing environmental risks of pesticides and developing coordinated management strategies that conserve natural enemies.

Glutathione S-transferases (GSTs, EC 2.5.1.18) represent a multifunctional supergene family of enzymes that are widely distributed across living organisms (Sheehan et al., 2001; Zhou et al., 2013). In mammals, glutathione S-transferases (GSTs) are classified into cytosolic GSTs (encompassing seven families: Alpha, Mu, Omega, Pi, Sigma, Theta, and Zeta), mitochondrial GSTs (Kappa family), and microsomal GSTs (MAPEG family), based on structural and enzymological characteristics (Board and Menon, 2013). GSTs are involved in several biological functions, including pesticide metabolism, antioxidation, hormonal transport, and olfactory processing (Xu et al., 2016; Enayati et al., 2005; Ketterman et al., 2011; Durand et al., 2018).

Recent research has highlighted the role of GSTs in mediating insecticide resistance. For example, *BmGSTu2* in *Bombyx mori* is capable of conjugating organophosphate insecticides (Yamamoto and Yamada, 2016). Similarly, RNAi silencing of *LmGSTs3* in *Locusta migratoria* increased sensitivity to carbaryl, confirming its involvement in detoxification (Qin et al., 2012). GST genes have also been identified in arachnids such as *Metaseiulus occidentalis*, *Ixodes scapularis*, and *Tetranychus cinnabarinus* (Wu and Hoy, 2016; Reddy et al., 2011; Zhao et al., 2021). However, related research on spiders remains limited, with only 22 full-length GST genes identified in *Achaearanea tepidariorum* and 13 full-length GST genes reported in *Pardosa pseudoannulata*, and in *P. pseudoannulata*, both *PpGSTd3* and *PpGSTt1* have been shown to respond to exposure of deltamethrin and imidacloprid (Liu et al., 2019). In insects, the Delta and Epsilon families represent class-specific GSTs that are uniquely present in this group and play a critical role in conferring resistance to various pesticides (Shi et al., 2012). However, there is a lack of comprehensive research on GST genes belonging to the Theta family, and it remains unclear whether they also confer functional roles in pesticide resistance.

To elucidate the selection mechanisms of insecticides and provide a theoretical basis for the conservation of such natural enemies, this study focuses on the functional characterization of the GST gene *PaGSTt1*. Based on transcriptomic data, we cloned the full-length *PaGSTt1* cDNA and performed bioinformatics and phylogenetic analyses. We then investigated the spatiotemporal expression patterns of *PaGSTt1* during development and in different tissues, as well as its transcriptional response to deltamethrin exposure. Finally, we used RNAi to assess whether silencing *PaGSTt1* affects the spider’s susceptibility to deltamethrin. These results will contribute to understanding detoxification pathways in natural enemies and provide molecular evidence for the development of ecologically safe pest management strategies.

## 2 Materials and methods

### 2.1 Spider collection and breeding

A laboratory colony of *P. astrigera* was originally established from individuals collected in a wheat experimental field in Shanxi Province, China. They were reared individually in glass tubes (1.5 cm in diameter, 8 cm high) with moistened cotton for humidity. They were kept in an incubator (Percival, Perry, IO, United States) under controlled conditions: 26 °C ± 1 °C, 60% ± 10% relative humidity, and a 14:10 h light/dark photoperiod. The spiders were fed *Drosophila melanogaster* prior to the third instar and *Tenebrio molitor* thereafter.

### 2.2 Gene cloning and sequence analysis

Adult male and female *P. astrigera* individuals were used for gene cloning. Each specimen was fully ground into a fine powder under liquid nitrogen to ensure thorough tissue disruption. Total RNA was extracted using the UNIQ-10 column-based total RNA extraction kit (Sangon, Shanghai, China) following the manufacturer’s protocol. RNA quality and concentration were assessed using a nucleic acid/protein analyzer and agarose gel electrophoresis. Only RNA samples with A260/A280 ratios between 1.9 and 2.0 were retained and stored at −80 °C for downstream applications.

First-strand cDNA was synthesized from high-quality RNA using the HiScript® II 1st Strand cDNA Synthesis Kit (Vazyme, Nanjing, China), and the open reading frame (ORF) of *PaGSTt1* was amplified by polymerase chain reaction (PCR). Specific primers were designed based on the *P. astrigera* transcriptome data using Primer Premier 5.0 software and synthesized by Shanghai Sangon Biotech Co., Ltd. The primer sequences are listed in Table 1.

**TABLE 1:** Primers used in this study.

	Primer sequence (5′-3’)	Use
*PaGSTt1*	F: ACTGAGATAGTGGCTAACTGA	PCR
	R: AAAGGCACTGGTTTGATTGTT	
q*PaGSTt1*	F: ATGATTTGATGTCACAGCCAT	RT-qPCR
	R: AGGCATAAAATGTTCCCCTTT	
*β-Actin*	F: GCAATCCTTCGTTTGGACTT	
	R: TTCTCTTTCAGCAGTGGTAGTGA	
ds*PaGSTt1*	F: TAATACGACTCACTATAGGG TACGCGTTTATTTGGTAGCAT	RNAi
	R:TAATACGACTCACTATAGGG CTGATGAACCTCACTATAATATGG	
ds*GFP*	F: TAATACGACTCACTATAGGG TCAGTGGAGAGGGTGAAGGT	
	R: TAATACGACTCACTATAGGG TGATGCCATTCTTTGGTTTG	

The PCR amplification was conducted in a 50 μL reaction system containing:2 μL of cDNA template, 1 μL of forward primer, 1 μL of reverse primer, 10 μL of 5× TransStart® FastPfu Buffer,1 μL of TransStart® FastPfu DNA Polymerase, 4 μL of dNTPs (2.5 mmol/L), 31 μL of nuclease-free water. The thermal cycling conditions were as follows: Initial denaturation at 95 °C for 1 min, 40 cycles of: 95 °C for 20 s, 56.5 °C for 20 s, and 72 °C for 1 min, final extension at 72 °C for 5 min.

PCR products were electrophoresed on an agarose gel, and the target bands were excised and purified. Purified fragments were ligated into vectors and transformed into competent cells. Positive colonies were verified by colony PCR and subsequently sent to Shanghai Sangon Biotech Co., Ltd. for Sanger sequencing. The T7 promoter sequence was underlined in all primer designs for dsRNA synthesis.

Conserved structural domains of the deduced amino acid sequence were analyzed using NCBI’s CD-Search tool (https://www.ncbi.nlm.nih.gov/Structure/cdd/wrpsb.cgi). The basic physicochemical properties of the *PaGSTt1* protein, including molecular weight, theoretical isoelectric point (pI), instability index, aliphatic index, and grand average of hydropathicity (GRAVY), were predicted using ExPASy ProtParam (https://web.expasy.org/protparam).

Hydrophilicity analysis was conducted using ExPASy ProtScale (https://www.expasy.org/tools/protscale.html). The presence of a signal peptide and its cleavage site were predicted using SignalP 5.0 (http://www.cbs.dtu.dk/services/SignalP/). Subcellular localization of the *PaGSTt1* protein was predicted using Cell-PLoc 2.0 (http://www.csbio.sjtu.edu.cn/bioinf/Cell-PLoc-2/).

Multiple sequence alignments were performed using ClustalX, and the results were visualized and annotated for secondary structure features using ESPript 3.0 (https://espript.ibcp.fr/ESPript/cgi-bin/ESPript.cgi). A phylogenetic tree was constructed using the neighbor-joining (NJ) method in MEGA 6.0 software, with 1,000 bootstrap replicates to assess branch reliability.

### 2.3 Detection of the spatiotemporal expression pattern of the *PaGSTt1* gene in *P. astrigera*

A total of 20 male and 20 female adults *P. astrigera* were anesthetized using liquid nitrogen, then cephalothorax, abdomen, and legs were quickly dissected on ice, and tissues of the same type were pooled into 1.5 mL centrifuge tubes and stored at −80 °C until use. For developmental stage analysis, 60 second-instar, 35 third-instar, 20 fourth-instar, 10 fifth-instar, 4 sixth-instar nymphs, 4 adult females and 4 adult males, were collected and pooled into separate centrifuge tubes and preserved at −80 °C. Each sample group was prepared in triplicate as biological replicates, three technical replicates were performed for each biological replicate. β-Actin was selected as the reference gene based on its previously demonstrated stable expression under insecticide-induced stress and RNAi treatment conditions in related arthropod models (Cao, 2024; Zhai et al., 2025).

Total RNA was extracted from each sample using a standard protocol and reverse-transcribed into first-strand cDNA using the HiScript II 1st Strand cDNA Synthesis Kit (Vazyme, Nanjing, China). The housekeeping gene *β-Actin* was used as an internal control for normalization (Li et al., 2016). Specific primers for the *PaGSTt1* gene and *β-Actin* were designed using Primer Premier 5.0 software and synthesized by Shanghai Sangon Biotech Co., Ltd. Primer sequences are listed in Table 1.

Quantitative real-time PCR (RT-qPCR) was performed using the ChamQ Universal SYBR qPCR Master Mix Kit (Vazyme, Nanjing, China) on a real-time PCR system. Each 20 μL reaction mixture contained: 10 μL of 2× ChamQ Universal SYBR qPCR Master Mix, 0.4 μL each of forward and reverse primers, 1 μL of cDNA template, and 8.2 μL of nuclease-free water.

The amplification program was as follows: Initial denaturation at 95 °C for 30 s; Followed by 40 cycles of denaturation at 95 °C for 10 s and annealing/extension at 60 °C for 30 s; Melting curve analysis was conducted at 95 °C for 15 s, 60 °C for 1 min, and 95 °C for 15 s. The specificity of amplification was confirmed by the presence of a single peak in the melting curve analysis.

### 2.4 The molecular docking of *PaGST* with deltamethrin

The 3D structure of *PaGSTt1* was predicted by homology modeling using the online server trRosetta (https://yanglab.qd.sdu.edu.cn/trRosetta/). The protein model quality was assessed by SAVES 6.0 (https://saves.mbi.ucla.edu/). Potential ligand-binding pockets were predicted with DoGSiteScorer (https://proteins.plus/).

Small molecule ligands were downloaded in SDF format from PubChem (https://pubchem.ncbi.nlm.nih.gov/) and converted to PDB format using Open Babel (3.1.1, Open Source Software, OSS) software. Hydrogen atoms were added to the ligands using AutoDock (4.2.6, Open Source Software, OSS) software (Morris et al., 2009). The protein structure was prepared by removing water molecules, adding hydrogens, and saving in pdbqt format. Semi-flexible docking was performed between the prepared ligands and protein. The docking results were visualized and analyzed using Discovery Studio (4.5, BIOVIA, San Diego, CA, United States) software (Wu et al., 2003).

Docking validation of the pre-built deltamethrin molecular model with the substrate CDNB (1-Chloro-2,4-dinitrobenzene) was performed, and the results are shown in Supplementary Table S5.

### 2.5 Detection of the expression level of *PaGSTt1* gene after deltamethrin stress

The concentrations of deltamethrin used in this study were based on previous toxicity bioassay results conducted by our research group on *P. astrigera*: LC_10_ = 5.151 mg/L, LC_30_ = 8.619 mg/L, and LC_50_ = 12.311 mg/L (Li, 2017). Adult male *P. astrigera* individuals with consistent body size and normal development were selected for the experiments. Deltamethrin exposure was performed using the film contact method as previously described (Chi et al., 2009).

A commercial deltamethrin stock solution (25 g/L) was diluted with acetone to the desired LC_10_, LC_30_, and LC_50_ concentrations for the treatment groups. Pure acetone was used as the control. One milliliter of each diluted solution was added to a clean 4 mL polypropylene tube. After gently shaking to coat the interior evenly, the solvent was allowed to evaporate naturally at room temperature, leaving a uniform insecticide film.

Each adult male spider was introduced into the treated tube and allowed to walk freely for 25 min to ensure cuticular contact. After exposure, the spiders were transferred into clean tubes and placed in an artificial climate incubator under standard rearing conditions. Specimens were collected at 6 h, 12 h, 18 h, 24 h, and 48 h post-exposure. At each time point, three individuals were sampled and immediately frozen in liquid nitrogen. Three biological replicates were performed for each time point and concentration, three technical replicates were performed for each biological replicate, resulting in a total of 225 treated spiders and 45 control spiders.

RNA extraction and first-strand cDNA synthesis were performed as described in Section 2.2. Quantitative real-time PCR (RT-qPCR) procedures, including reaction setup and cycling conditions, were identical to those outlined in Section 2.3.

### 2.6 Functional study of *PaGSTt1* gene in *P. astrigera*

Double-stranded RNAs (dsRNAs) targeting *PaGSTt1* (*dsPaGSTt1*) and a control green fluorescent protein gene (dsGFP) were synthesized using the T7 High Yield RNA Transcription Kit. Primer sequences used for dsRNA synthesis are listed in Table 1.

For RNAi delivery, a dsRNA–nanocarrier complex was prepared according to a standard dosage of 1 μg dsRNA per adult male *P. astrigera*. The dsRNA was mixed with the nanocarrier solution at a mass ratio of 1:1, incubated at room temperature for 15 min, and then 1% solution of liquid B was added to reduce surface tension. Using a microsyringe, a droplet of the mixture was topically applied to the dorsal surface of each spider.

To evaluate gene silencing efficiency, surviving *P. astrigera* individuals were collected at 3 h, 6 h, 12 h, 18 h, 24 h, and 48 h post-application and immediately frozen in liquid nitrogen. For each time point, three spiders were sampled, and the experiment was repeated in triplicate, three technical replicates were performed for each biological replicate. RNA extraction and cDNA synthesis were performed as described in Section 2.2, and RT-qPCR was conducted following the procedures in Section 2.3 to assess the silencing efficiency of *PaGSTt1* expression.

At the time point showing the highest gene silencing efficiency, deltamethrin at LC_30_ concentration was employed to treat the RNAi-treated spiders using the film contact method, following the same procedure outlined in Section 2.5. Both treatment and control groups were established, the control group includes treatments with H_2_O only, with the nanomaterial (SPc) only, and with *dsGFP*. Each group consisted of 30 male adult spiders, and three independent biological replicates were conducted for this study, each using an independently cultured population. Spider survival was assessed at 48 h post-treatment, and recorded the number of deaths.

### 2.7 Statistical analysis

Experimental data were analyzed using SPSS 27.0 .1.0(IBM, Armonk, NY, United States) with single factor ANOVA test, independent-sample t-tests, and chi-square. Relative gene expression levels were calculated using the 2^−ΔΔCT^ method (Livak and Schmittgen, 2001). Graphs were visualized using Origin 2021 (OriginLab, Northampton, MA, United States) software.

## 3 Results

### 3.1 Cloning and sequence analysis of *PaGSTt1*

Based on the transcriptome data of *P. astrigera*, the full-length cDNA of the *PaGSTt1* gene was successfully cloned. The sequence is 824 bp in length and includes a complete open reading frame (ORF) of 678 bp, encoding a protein of 225 amino acids. The predicted molecular formula of the protein is C_1210_H_1901_N_309_O_333_S_9_, with a molecular weight of 26.39 kDa and an isoelectric point (pI) of 8.79. The instability index was calculated as 37.41, indicating that the protein is stable. The grand average of hydropathicity (GRAVY) was −0.305, suggesting that *PaGSTt1* is a hydrophilic protein.

**FIGURE 1:**
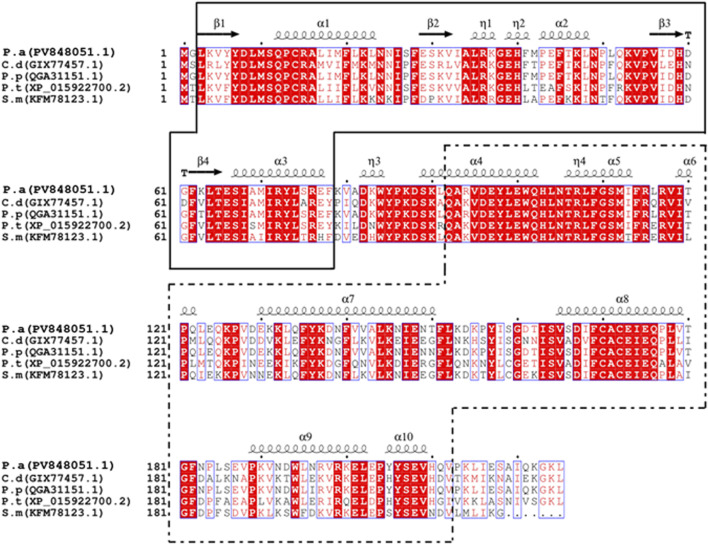
Comparison of GST amino acid sequences between *PaGSTt1* of *P. astrigera* and other species. C.d: *Caerostris darwini*;P.p: *Pardosa pseudoannulata*;P.t:*Parasteatoda tepidariorum*;S.m: *Stegodyphus mimosarum*;P.a: *Pardosa astrigera*. The blacksolid frame is the complete N-terminal domain of *PaGSTt1*, and the black dotted frame is the complete C-terminal domain ofPaGSTt1. α-helix and β-folds are automatically added by the ESPriptonline tool.

Subcellular localization analysis predicted that *PaGSTt1* is a cytoplasmic GST and lacks a signal peptide, classifying it as a non-secretory protein. Conserved domain analysis indicated that *PaGSTt1* contains the GST-N-Theta (residues 3–78) and GST-C-Theta (residues 92–212) domains. Multiple sequence alignment with GSTs from other arachnid species confirmed the conservation of these domains. The N-terminal region of *PaGSTt1* exhibits a typical β–α–β–α–β–α structure, whereas the C-terminal region consists entirely of α-helices, lacking β-strands (Figure 1).

**FIGURE 2:**
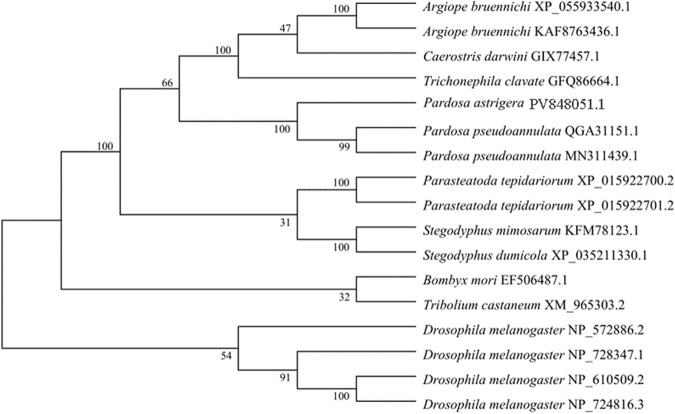
Phylogenetic tree of *PaGSTt1* with GSTs of other species constructed by neighbour-joining method.

GST protein sequences from various species were retrieved from the NCBI database and used to construct a phylogenetic tree together with the *P. astrigera PaGSTt1* protein using the neighbor-joining (NJ) method. The clustering analysis indicated that *PaGSTt1* grouped closely with other arachnid GST proteins. Notably, *PaGSTt1* demonstrated the highest sequence similarity to *Pardosa pseudoannulata*, with 98.22% identity to the QGA31151.1 protein sequence (Figure 2). This close evolutionary relationship supports the conserved nature of GST proteins among related arachnid species.

### 3.2 Spatiotemporal expression of *PaGSTt1* gene

**FIGURE 3:**
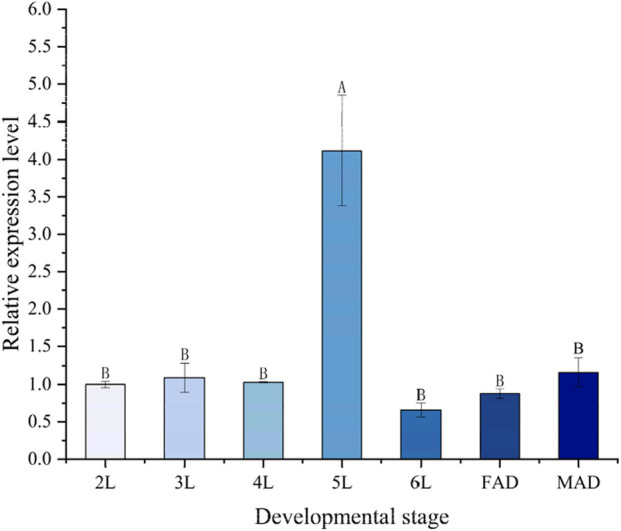
Relative expression level of *PaGSTt1* gene in *P. astrigera* at different developmental stages. 2L‒6L: 2nd‒6th instar nymphs; FAD: Female Adult; MAD: Male Adult. Data in the figure are mean ± *SD*. Different capital letters above bars indicate significant difference in the gene expression level among different developmental stages by Duncan’s new multiple range test (*P* < 0.05).

The relative expression levels of the *PaGSTt1* gene at different developmental stages of *P. astrigera* are shown in Figure 3. *PaGSTt1* was expressed at all examined stages, from the 2nd instar nymph to the adult stage. Among these, the expression level was significantly highest in the 5th instar nymph (p < 0.05), suggesting a potential developmental peak in gene activity. In contrast, the lowest expression was observed in the 6th instar nymph, with no statistically significant differences compared to the 2nd, 3rd, and 4th instar nymphs or the adult stage (p > 0.05). These findings indicate that *PaGSTt1* may play a critical role during the mid-larval stage of development.

**FIGURE 4:**
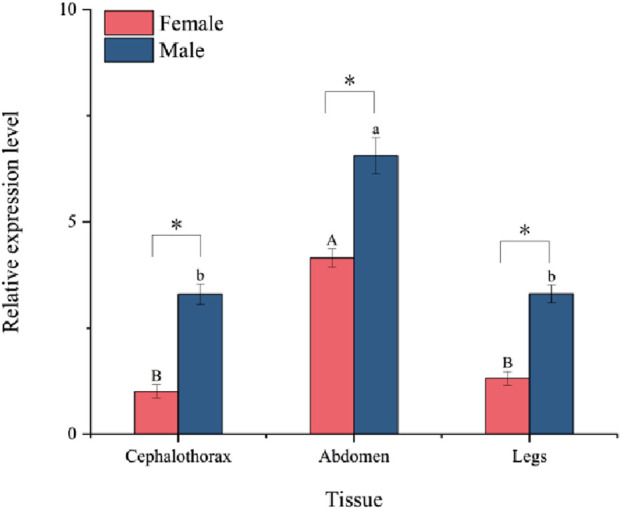
Relative expression of *PaGSTt1* in different tissues of male and female adult *P. astrigera*.

The relative expression levels of the *PaGSTt1* gene in different tissues (cephalothorax, abdomen, and legs) of male and female adult *P. astrigera* are presented in Figure 4. *PaGSTt1* was expressed in all examined tissues, with significant differences observed among them (p < 0.05). In both sexes, the expression level of *PaGSTt1* was highest in the abdomen, which was significantly higher than in the cephalothorax and legs (p < 0.05). Furthermore, in each corresponding tissue, male spiders exhibited significantly higher expression levels of *PaGSTt1* compared to females (p < 0.05). These results suggest a possible sex-biased and tissue-specific regulation of *PaGSTt1*, with the abdomen being the primary site of gene activity.

Data in the figure are mean ± *SD*. Different uppercase and lowercase above bars indicate significant difference in the gene expression level among different tissues of female and male adults, respectively, by using single factor ANOVA test followed by Tamhane’s T2 multiple comparisons, a value of p < 0.05 was considered statistically significant. Symbols above bars indicate significant difference in the gene expression level in the same tissue between different genders by independent samples t-test (*P < 0.05).

### 3.3 *PaGSTt1* in *P. astrigera* molecular docking with deltamethrin

**FIGURE 5:**
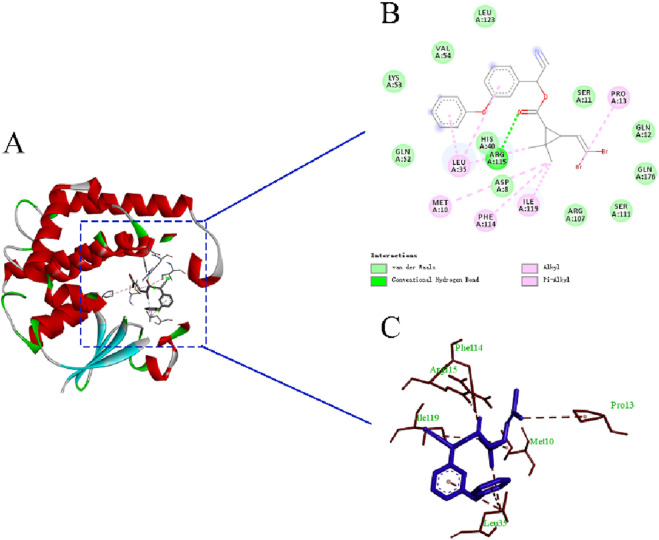
The binding model of *PaGSTt1* with deltamethrin. Note: **(A)** Combined appearance pattern diagram; **(B)** Combined two-dimensional structure diagram; **(C)** Combined three-dimensional structure.

The molecular docking results and the binding model between *PaGSTt1* and deltamethrin are shown in Table 2 and Figure 5. The binding energy of *PaGSTt1* with deltamethrin was −7.56-kcal/mol. The amino acid residue ARG (A:115) formed a hydrogen bond with the protein, while 11 amino acid residues—ASP(A:8), SER(A:11), GLN (A:12), HIS(A:40), GLN (A:52), LYS(A:53), VAL(A:54), ARG (A:107), SER(A:111), LEU (A:123), and GLN (A:176)—participated in hydrophobic interactions. Additionally, five amino acid residues—MET (A:10), PRO (A:13), LEU (A:35), PHE(A:114), and ILE (A:119)—were involved in Van der Waals forces.

**TABLE 2:** Molecular docking results of *PaGSTt1* and deltamethrin from *P. astrigera*.

Gene name	Macromolecule ligands	CAS number	Hydrogen bond formation site	Hydrophobic interaction formation site	Van der waals force formation site	Binding energy (kcal/mol)
*PaGSTt1*	deltamethrin	52918-63-5	ARG (A:115)	ASP(A:8), SER(A:11), GLN (A:12), HIS(A:40), GLN (A:52), LYS(A:53), VAL(A:54), ARG (A:107), SER(A:111), LEU (A:123), GLN (A:176)	MET (A:10), PRO (A:13), LEU (A:35), PHE(A:114), ILE (A:119)	−7.56

### 3.4 Expression patterns of *PaGSTt1* gene in *P. astrigera* after deltamethrin treatment

**FIGURE 6:**
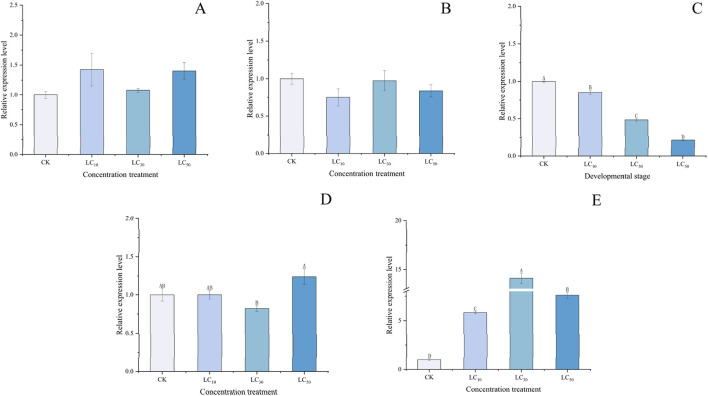
Relative expression level of *PaGSTt1* gene in male adult *P. astrigera* treated with different concentrations of deltamethrin. CK: Equal acetone treatment as the control. LC_10_: 5.151 mg/L; LC_30_: 8.619 mg/L; LC_50_: 12.311 mg/L. A: 6 h, B: 12 h, C: 18 h, D:24 h, E:48 h. Data in the figure are mean ± SD, use calculated using single factor ANOVA test followed by Tukey’s HSD multiple comparisons, a value of p < 0.05 was considered statistically significant. Different letters above bars indicate significant difference in the gene expression level among different treatments.

Significant differences were observed in the relative expression of the *PaGSTt1* gene in male adult *P. astrigera* following exposure to different concentrations of deltamethrin at various time points (Figure 6).

At different time points, there were no significant differences (p > 0.05) in the relative expression of *PaGSTt1* between various concentrations of deltamethrin treatments and the control (acetone) at 6, 12, and 24 h (Figures 6A,B,D). At 18 h (Figure 6C), the relative expression levels in all treatment groups were lower than those in the control (acetone), with higher concentrations resulting in progressively lower expression levels, and significant differences (p < 0.05) observed between each treatment group. At 48 h (Figure 6E), the relative expression levels in all treatment groups were higher than those in the control (acetone), with the LC_30_ group showing the highest expression and the LC_10_ group the lowest, along with significant differences (p < 0.05) between each treatment group. These results indicate that *PaGSTt1* exhibits a complex, time-dependent, and dose-dependent regulatory response to deltamethrin stress in *P. astrigera*.

### 3.5 Functional analysis of *PaGSTt1* gene using RNAi

**FIGURE 7:**
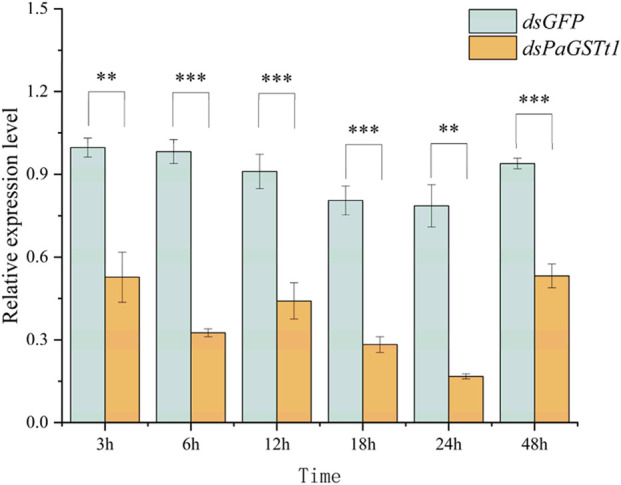
Expression of *PaGSTt1* gene in *P. astrigera* after dsRNA injection for different time Data are presented as mean ± SD. Independent sample t-test p-value, significant differences are indicated by asterisks (**: p < 0.01, ***: p < 0.001).

The efficiency of RNAi was evaluated by measuring *PaGSTt1* gene expression at 3, 6, 12, 18, 24, and 48 h post-dsRNA treatment using RT-qPCR, with dsGFP serving as the control at each time point (Figure 7). The results demonstrated that at all six time points, the expression level of *PaGSTt1* was significantly reduced compared to the control group (p < 0.05). The most effective gene silencing occurred at 24 h, where *PaGSTt1* expression was decreased to 21.31% of the control level, representing a 78.69% knockdown. However, at 48 h, *PaGSTt1* expression partially rebounded. No significant differences were found between the 3 h and 12 h groups (p > 0.05).

**FIGURE 8:**
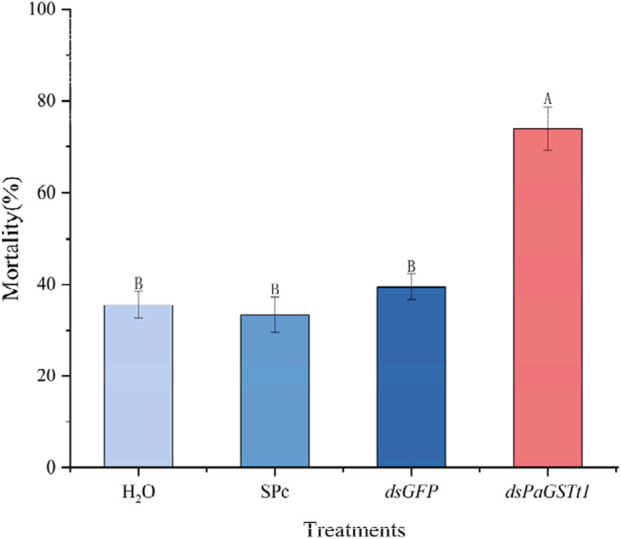
The mortality rate of *P. astrigera* after RNAi. Data in the figure are mean ± SD, use calculated using single factor ANOVA test followed by Tukey’s HSD multiple comparisons, a value of p < 0.05 was considered statistically significant. Different letters above bars indicate significant difference in the gene expression level among different treatments (n = 3 independent biological replicates, each consisting of tissue pooled from 30 spiders).

To assess the impact of *PaGSTt1* silencing on susceptibility to deltamethrin, adult male *P. astrigera* were exposed to LC_30_ deltamethrin at the optimal silencing time point (24 h post-dsRNA treatment), using the same drug film application method described previously. The mortality rate of the RNAi-treated group was significantly higher than that of the control group (p < 0.05) (Figure 8), with 73.98% mortality in the treatment group—an increase of 34.54% compared to controls (*dsGFP*).

These findings indicate that RNAi-mediated knockdown of *PaGSTt1* increases the sensitivity of *P. astrigera* to deltamethrin, suggesting that *PaGSTt1* plays a role in the detoxification and metabolism of deltamethrin in this species.

## 4 Discussion

Glutathione S-transferases (GSTs) are pivotal detoxification enzymes that facilitate the conjugation of glutathione to a variety of endogenous and exogenous compounds, thereby enhancing their solubility and excretion. In arthropods, GSTs play a crucial role in metabolizing xenobiotics, including insecticides, and in mitigating oxidative stress induced by these compounds (Xu et al., 2025; Ma et al., 2024; Hu et al., 2022; Hu et al., 2014). The spider *P. astrigera*, a natural predator in agricultural ecosystems, is instrumental in controlling pest populations and is consequently exposed to various insecticides. Understanding the detoxification mechanisms in *P. astrigera* is essential for assessing the ecological impact of pesticide use and for developing sustainable pest management strategies.

In this study, we identified and characterized a GST gene, *PaGSTt1*, from *P. astrigera*. The full-length cDNA sequence of *PaGSTt1* is 824 bp, encompassing an open reading frame of 678 bp that encodes a 225-amino acid protein. Sequence analysis revealed conserved domains characteristic of the Theta class GSTs, including the N-terminal GSH-binding site (G-site) and the C-terminal hydrophobic substrate-binding site (H-site). Phylogenetic analysis indicated that *PaGSTt1* shares high sequence similarity with GSTs from other arachnid species, particularly *Pardosa pseudoannulata*, suggesting evolutionary conservation of function.

Tissue-specific expression analysis demonstrated that *PaGSTt1* is differentially expressed across various tissues, with the highest expression observed in the abdomen of both male and female adult spiders. This region houses key detoxification organs such as the midgut, fat body, and Malpighian tubules, implicating *PaGSTt1* in the metabolic processing of xenobiotics. Furthermore, developmental stage analysis indicated elevated expression of *PaGSTt1* during the fifth instar nymph stage, a period associated with increased feeding activity and, consequently, higher exposure to dietary toxins. These findings align with studies in other arthropods where GST expression correlates with developmental stages and tissue types involved in detoxification (Zhang et al., 2024; Yang et al., 2025; Enders et al., 2020).

It is noteworthy that the expression of *PaGSTt1* under deltamethrin stress demonstrated an initial downregulation followed by upregulation. Its significant overexpression at 48 h is consistent with our earlier findings on GST enzyme activity assays (Supplementary Table S6). We speculate that *PaGSTt1* is a key GST gene involved in the metabolism of deltamethrin in *P. astrigera*. The involvement of GST genes in detoxification may rely on a complex system, where different genes perform distinct functions at different stages. Han et al. (2016) hypothesized that the downregulation of GST genes could be a stress response, and the upregulation of a large number of GST genes may require the downregulation of some genes to avoid excessive depletion of reduced glutathione in insects. Similar inducible expression of GSTs in response to insecticide exposure has been documented in various insect species, including *Bactrocera dorsalis*, *Nilaparvata lugens*, and *Sesamia inferens* (Li et al., 2024; Gao et al., 2021; Fang et al., 2008).

Molecular docking analysis suggests a potential binding affinity between deltamethrin and the active site of *PaGSTt1*. This predicted interaction may potentially explain the observed metabolic fate of deltamethrin in *P. astrigera*. However, it must be emphasized that docking results only indicate a possibility of binding and do not constitute proof of enzymatic activity or metabolic function. Further functional validation is required to confirm this hypothesized interaction and its catalytic consequences.

To elucidate the functional role of *PaGSTt1* in deltamethrin detoxification, RNAi was employed to silence gene expression. Quantitative RT-PCR confirmed significant knockdown of *PaGSTt1* expression at multiple time points post-dsRNA administration, with the most pronounced suppression (78.69% reduction) observed at 24 h. Subsequent bioassays indicated that *PaGSTt1*-silenced spiders exhibited a 34.54% increase in mortality upon deltamethrin exposure compared to controls, indicating heightened sensitivity due to impaired detoxification capacity. These findings corroborate previous studies where RNAi-mediated silencing of GST genes in insects led to increased susceptibility to insecticides. The inferred dsRNA delivery efficiency in this study is based on prior characterization of this nanocarrier (SPc) in insects (Wang, 2022). Future studies could further validate its uptake kinetics in *P. astrigera* through methods such as fluorescence imaging experiments.

Although this study demonstrates that the expression of *PaGSTt1* (a Theta-class GST) is significantly upregulated under deltamethrin stress, and its temporal dynamics align with the changes in total GST enzyme activity—suggesting its potential involvement in detoxification metabolism—a cautious interpretation of its specific function is warranted. As reviewed by Hu et al. (2019), insect-specific Delta and Epsilon class GSTs are primarily associated with insecticide metabolism and resistance. In contrast, the primary physiological role of Theta-class GSTs may lean more towards scavenging toxic secondary products generated by oxidative stress rather than directly metabolizing insecticides themselves. Therefore, the observed upregulation of *PaGSTt1* at 48 h could represent a compensatory response to oxidative stress induced by deltamethrin, indirectly contributing to resistance by protecting cells from oxidative damage. This hypothesis offers a novel perspective, distinct from the traditional metabolic detoxification model, for explaining Theta-class GST involvement in resistance.

While this study preliminarily reveals the potential role of *PaGSTt1* in responding to deltamethrin stress, several limitations must be acknowledged. First, although RNAi can induce phenotypic changes (such as mortality), it cannot fully distinguish whether the gene is directly involved in detoxification metabolism or indirectly affects overall physiological status (such as antioxidant capacity). Second, this study lacks *in vitro* biochemical validation of the direct metabolic capacity of the *PaGSTt1* protein against deltamethrin.

To further elucidate the function of *PaGSTt1,* future research should include:

A. Indoor selection of a deltamethrin-resistant strain of *P. astrigera* and comparison of *PaGSTt1* expression levels (Zhu et al., 2002; Li et al., 2020), GST enzyme activity, and metabolic rates between resistant and susceptible strains.
B. Heterologous expression and purification of recombinant *PaGSTt1* protein to directly measure the kinetic parameters of its catalysis of deltamethrin metabolism (Hirowatari et al., 2017; Xu et al., 2023), providing decisive evidence for its direct metabolic capability.
C. Utilization of CRISPR/Cas9 gene editing technology (Jin et al., 2023; Xiao et al., 2025) to generate a *PaGSTt1* knockout strain, enabling validation of its function by comparing differences in insecticide susceptibility, metabolite accumulation, and oxidative stress levels between knockout mutants and wild-type individuals.

## Data Availability

The original contributions presented in the study are publicly available. This data can be found here: The Gene PaGSTt1 has been uploaded to the NCBI database under GenBank accession number PV848051.1.
