# Supplementary material for: RNAi-based functional analysis of a theta-class glutathione S-transferase implicated in deltamethrin detoxification in Pardosa astrigera (Araneae: Lycosidae)
Source: Front Physiol. 2025 Oct 15;16:1693654. doi: 10.3389/fphys.2025.1693654 (PMC12562982; doi:10.3389/fphys.2025.1693654)
Supplement: Supplementary file 1 [file Table1.docx]

**Table S1.** The sequence information of *PaGSTt1*

| *PastGSTt1* Nucleotide | ATTTTAATCATAAACACGTAGATATTAATCTCGGGTTGCGATAGAATCAACGAATCGTAAGGTTACATGATTATTTACCCAAGAAATCAAATTTTTGAGTGTAGTCACGAGGTTTTAAACACTGAGATAGTGGCTAACTGAAATCCTTCTACGGAAGATAGAGTACATTTTTTAAGGATATATATATATTTACATTTCATTATTACCTAAAAATGGGTTTAAAAGTATATTATGATTTGATGTCACAGCCATGTCGTGCCTTAATAATGTTTCTTAAACTGAATAATATTTCATTTGAATCCAAAGTTATTGCATTAAGAAAAGGGGAACATTTTATGCCTGAGTTTACTAAATTGAATCCTCTTCAGAAAGTACCTGTTATTGATCATGATGGGTTTAAGTTAACTGAAAGTATTGCAATGATACGATATTTGTCAAGAGAGTTTAAAGTGGCAGATAAGTGGTATCCTAAAGACAGTAAACTTCAAGCAAGAGTGGATGAATATTTAGAGTGGCAACACCTGAATACGCGTTTATTTGGTAGCATGATATTTCGCCTTAGGGTTATTACCCCACAGTTGGAACAAAAACCAGTTGATGAAAAAAAGTTGCAGTTTTATAAGGATAATTTTGTAGTTGCATTGAAAAACATTGAAAACACCTTTTTGAAAGATAAACCCTATATCAGTGGTGATACTATATCTGTCTCTGATATATTTTGTGCATGTGAAATTGAACAGCCATTGGTTACTGGCTTTAATCCATTGTCTGAAGTTCCTAAAGTTAATGATTGGTTAAATAGAGTCAGAAAGGAACTTGAACCATATTATAGTGAGGTTCATCAGGTACCTAAGTTAATTGAAAGTGCAATTCAGAAAGGAAAACTGTAAAATAGTTTATTTTCTATCTTTGAGTTAAAAAATAACAATCAAACCAGTGCCTTTTTCATTATAAATAATATACAAATCAAGCATTTATAGTATGTATTATGGTATTATACTTTAATAAAACTTTGATTTTTCTTTCAAAAAAA |
| --- | --- |
| *PaGSTt1*  Protein | MGLKVYYDLMSQPCRALIMFLKLNNISFESKVIALRKGEHFMPEFTKLNP  LQKVPVIDHDGFKLTESIAMIRYLSREFKVADKWYPKDSKLQARVDEYLE  WQHLNTRLFGSMIFRLRVITPQLEQKPVDEKKLQFYKDNFVVALKNIENT  FLKDKPYISGDTISVSDIFCACEIEQPLVTGFNPLSEVPKVNDWLNRVRK  ELEPYYSEVHQVPKLIESAIQKGKL |

**Table S2.** The sequence information of *dsPaGSTt1*

|  | **Sequence information** | **Length** | **GC content** |
| --- | --- | --- | --- |
| *dsPaGSTt1* | TACGCGTTTATTTGGTAGCATGATATTTCGCCTTAGGGTTATTACCCCACAGTTGGAACAAAAACCAGTTGATGAAAAAAAGTTGCAGTTTTATAAGGATAATTTTGTAGTTGCATTGAAAAACATTGAAAACACCTTTTTGAAAGATAAACCCTATATCAGTGGTGATACTATATCTGTCTCTGATATATTTTGTGCATGTGAAATTGAACAGCCATTGGTTACTGGCTTTAATCCATTGTCTGAAGTTCCTAAAGTTAATGATTGGTTAAATAGAGTCAGAAAGGAACTTGAACCATATTATAGTGAGGTTCATCAG | 319bp | 34% |

**Table S3.** Stability of internal reference genes of P. astrigera based on NormFinder analysis.

| Genes | Ages | | Sexes | | | Female adult tissues | | | | Male adult tissues | |
| --- | --- | --- | --- | --- | --- | --- | --- | --- | --- | --- | --- |
|  | Stability value | Rank | | Stability value | Rank | | Stability  value | Rank | | Stability value | Rank |
| Hsc70 | 0.48 | 3 | 0.62 | | 5 | 0.39 | | | 4 | 0.49 | 4 |
| AK | 0.96 | 7 | 0.39 | | 4 | 1.4 | | | 7 | 1.4 | 8 |
| GAPDH | 0.5 | 4 | 0.65 | | 7 | 0.42 | | | 5 | 0.34 | 2 |
| α-Tub | 1.79 | 8 | 1.85 | | 8 | 0.38 | | | 2 | 0.83 | 5 |
| β-Actin | 0.23 | 2 | 0.19 | | 1 | 0.14 | | | 1 | 0.12 | 1 |
| SDHA | 0.16 | 1 | 0.37 | | 2 | 0.98 | | | 6 | 0.85 | 6 |
| RPL32 | 0.95 | 6 | 0.37 | | 3 | 2.1 | | | 8 | 1.31 | 7 |
| EF1-α | 0.54 | 5 | 0.62 | | 6 | 0.39 | | | 3 | 0.44 | 3 |

| **Binding energy distribution** | **Count** |
| --- | --- |
| -7.56 ~ -7.2 | 4 |
| -7.2 ~ -6.3 | 11 |
| -6.3 ~ -5.4 | 12 |
| -5.4 ~ -4.5 | 16 |
| -4.5 ~ -3.6 | 7 |

**Table S4.** The binding energy distribution obtained from AutoDock（n=50）

**Table S5.** The binding of two ligands to *PaGSTt1*（n=50）

| **Ligands** | **CAS number** | **AutoDock Vina score (kcal/mol) [Mean ± SD]** |
| --- | --- | --- |
| CDNB（1-Chloro-2,4-dinitrobenzene） | 97-00-7 | -6.218 ± 0.056 |
| Deltamethrin | 52918-63-5 | -5.77 ± 0.97 |

**Table S6.** Effects of low-dose of deltamethrin on activities of GSTs of Pardosa astrigera(U/mgprot)

|  | **24h** | **48h** |
| --- | --- | --- |
| **CK** | 23.502±0.55 | 43.914±1.16 |
| **LC10** | 49.525±1.115 | 63.603±0.58 |
| **LC30** | 67.071±1.40 | 80.535±1.44 |
| **LC50** | 52.429±1.23 | 52.162±0.68 |
